# Diabetes in Pregnancy and MicroRNAs: Promises and Limitations in Their Clinical Application

**DOI:** 10.3390/ncrna4040032

**Published:** 2018-11-12

**Authors:** Adriana Ibarra, Begoña Vega-Guedes, Yeray Brito-Casillas, Ana M. Wägner

**Affiliations:** 1 Endocrinology and Nutrition Department, Complejo Hospitalario Universitario Insular Materno-Infantil de Gran Canaria, 35016 Las Palmas de Gran Canaria, Spain; aibarraggg@gmail.com; 2 Instituto de Investigaciones Biomédicas y Sanitarias (IUIBS), Universidad de Las Palmas de Gran Canaria, 35016 Las Palmas de Gran Canaria, Spain; begovegag@hotmail.com (B.V.-G.); yeray.brito@ulpgc.es (Y.B.-C.); 3 Obstetrics and Gynaecology Department, Complejo Hospitalario Universitario Insular Materno-Infantil de Gran Canaria, 35016 Las Palmas de Gran Canaria, Spain

**Keywords:** intrauterine programming, pre-gestational diabetes, gestational diabetes, diabetic embryopathy, macrosomia

## Abstract

Maternal diabetes is associated with an increased risk of complications for the mother and her offspring. The latter have an increased risk of foetal macrosomia, hypoglycaemia, respiratory distress syndrome, preterm delivery, malformations and mortality but also of life-long development of obesity and diabetes. Epigenetics have been proposed as an explanation for this long-term risk, and microRNAs (miRNAs) may play a role, both in short- and long-term outcomes. Gestation is associated with increasing maternal insulin resistance, as well as β-cell expansion, to account for the increased insulin needs and studies performed in pregnant rats support a role of miRNAs in this expansion. Furthermore, several miRNAs are involved in pancreatic embryonic development. On the other hand, maternal diabetes is associated with changes in miRNA both in maternal and in foetal tissues. This review aims to summarise the existing knowledge on miRNAs in gestational and pre-gestational diabetes, both as diagnostic biomarkers and as mechanistic players, in the development of gestational diabetes itself and also of short- and long-term complications for the mother and her offspring.

## 1. Introduction

### 1.1. Diabetes in Pregnancy: Classification

Pre-gestational diabetes, usually type 1 or type 2, diagnosed before conception, is present in approximately 1% of all pregnancies [1]. Gestational diabetes is defined as impaired glucose tolerance diagnosed, for the first time, during pregnancy and is present in 6–7% of pregnancies [1]. The prevalence of pregnancy complicated by diabetes (gestational or pre-gestational) varies geographically and among different ethnic groups. Its recent increase has been attributed to increasing maternal age and, especially, to the increasing prevalence of obesity [1,2].

During normal pregnancy, a series of metabolic changes take place. There is a progressive increase in insulin resistance, mainly due to placental hormone production (growth hormone, corticotropin, placental lactogen and progesterone), which plays a crucial role in foetal nutrition. Gestational diabetes is believed to develop in women whose insulin production is insufficient to counteract increasing insulin resistance (reviewed in Reference [3]), although some metabolic heterogeneity has been described when comparing women with gestational diabetes to controls [4].

All pregnant women should be screened for gestational diabetes mellitus (GDM) with a laboratory-based screening test using blood glucose levels [5]. Screening for GDM is usually performed at 24–28 weeks of gestation. Nevertheless, there is no consensus regarding the test to use. The most commonly used is a two-step test, the first step being the administration of a 50 g oral glucose solution, followed by a 1 h venous glucose measurement. Women whose glucose levels meet a threshold then undergo a 100 g, 3 h diagnostic oral glucose tolerance test. Different cut-offs have been proposed for the 3 h test (see Table 1). In the absence of clear comparative trials, one set of diagnostic criteria for the 3 h test cannot be clearly recommended over the other (reviewed in Reference [5,6,7]).

A one-step test using a 75 g, 2 h oral glucose test was initially proposed by the International Association of Diabetes and Pregnancy Study Group [8] and then adopted by the American Diabetes Association [9] (see Table 1) but there is lack of evidence that the use of the one-step test leads to clinically significant improvements in maternal or newborn outcomes [10,11].

When diagnosed, gestational diabetes that is adequately controlled without medication is often classified as diet-controlled or class A1 gestational diabetes, as opposed to that requiring medication to achieve euglycemia, which is classified as A2 [5].

### 1.2. Clinical Consequences of Diabetes in Pregnancy

Pregnancies which are complicated with diabetes are associated with a higher risk of poor outcomes, both for the mother and the offspring. Indeed, pregnancy outcomes are generally correlated with the severity of maternal diabetes, that is, complications are more frequent and severe when maternal diabetes is more severe and worse controlled [12]. Women with gestational diabetes can have similar types of complications to those of women with pre-gestational diabetes, although individual risk is lower, due to the fact that gestational diabetes is a milder metabolic disorder than pre-gestational diabetes. Hyperglycaemia is the main cause of this increased risk and improved glycaemic control before (in pre-gestational diabetes) and during pregnancy is associated with improved outcomes [12,13].

Maternal risks include hypoglycaemia in the beginning of pregnancy, diabetic ketoacidosis and progression of retinopathy or nephropathy. Women with gestational diabetes have an increased risk of developing diabetes in the future [14].

Pregnancy and foetal risks include abortions, congenital malformations, preterm delivery, preeclampsia, caesarean section, foetal macrosomia, neonatal hypoglycaemia, hyperbilirubinemia, shoulder dystocia, birth trauma and stillbirth [15,16]. Long-term studies also show that exposure to intrauterine hyperglycaemia is associated with an increased risk of obesity, glucose intolerance and diabetes [17,18,19].

### 1.3. Epigenetics and Intrauterine Programming

There is strong evidence that maternal nutrition and lifestyle during pregnancy have persisting effects on the health of the offspring (reviewed in Reference [20]). The concept “Developmental Origins of Health and Disease” refers to the consequences early, unfavourable factors can have on future, life-time risk of disease. Although controversial [21,22], epigenetics have been proposed as an explanation for the increased life-long risk of diabetes and obesity in the offspring of mothers with diabetes [23,24] or obesity [25]. Indeed, extensive epigenetic reprogramming is known to occur early in embryonic life [26,27] and the intra-uterine environment is a plausible candidate to modulate these effects.

Epigenetic changes regulate gene expression without a change in the nucleotide sequence, they are heritable, reversible and can be induced by environmental factors, thus having an impact on normal development and acquired traits and disorders. Epigenetic regulation primarily includes DNA methylation, histone modifications and non-coding RNA. DNA methylation, which is generally associated with gene silencing, has been the most studied epigenetic mark so far and its potential role in type 2 diabetes programming has been recently reviewed [23]. Histone modifications mainly determine accessibility of DNA to the transcription machinery [28], whereas non-coding RNAs are primarily posttranscriptional regulators. The present review specifically focuses microRNA (miRNA), short, non-coding RNAs, in gestational and pre-gestational diabetes. This classification of epigenetic mechanisms is merely academic, however, since all these regulatory systems act in an orchestrated manner [26,27]. Indeed, miRNA have shown regulatory effects both on histone modifications [29] and on DNA methylation (reviewed in Reference [30]).

## 2. MiRNAs

### 2.1. Pregnancy and miRNAs: The Role of the Placenta

The placenta is a mixed maternofoetal organ which provides support to the growing foetus from the maternal blood supply, in the form of nutrient uptake, waste elimination and gas exchange. It acts as an interface between the foetal umbilical cord and the maternal uterine wall and is highly adaptive to the needs of the growing foetus. It is also an endocrine organ, secreting placental lactogen, growth hormone, oestrogen, progesterone, chorionic gonadotrophin and neuroactive hormones, such as melatonin, serotonine and oxytocin, which play a central role in maternal physiological adaptation to pregnancy (reviewed in Reference [31]).

The human placenta is estimated to express 69% of all human proteins [32], although most of them act as housekeeping genes, given their stable expression. Those genes which are highly expressed in the placenta are mainly associated with pregnancy and with oestrogen biosynthetic and metabolic pathways [32]. The human placenta also expresses numerous types of miRNA species (reviewed in Reference [33]), which are encoded at all but chromosome Y [34]. Some have shown to be almost specific to the placenta (expressed in trophoblasts but also in the brain) and to vary from the first trimester to term [34]. Specific placental miRNAs include miR-512-3p, miR-517a, miR-517b, miR-518b and miR-519b, which belong to a cluster on chromosome 19 (C19MC) and can be detected in maternal blood (miR-517a) [34]. Another, more recently described, placenta-specific miRNA cluster is C14MC, which includes miR-127-3p, miR-370, miR-441, miR-539 and more than 40 others [35]. miRNAs on C19MC increase their expression at the end of pregnancy, whereas those on C14MC reduce their expression [35]. Indeed, miRNAs associated with pregnancy can be classified as placenta-specific, placenta associated, placenta-derived circulating and uterine microRNA, according to their localization and origin [36]. Studies performed in mice and mouse cell-lines support the role of miR-675, contained in the lncRNA H19, in the regulation of placental growth. Indeed, the expression of miR-675 increases at the end of pregnancy, as placental growth ceases [37]. Clinical applications of the study of (circulating) placental miRNA include prediction of pre-eclampsia, which is beyond the scope of this paper and has recently been reviewed [38,39].

Although results are not consistent among studies (reviewed in Reference [40]), in a recent report, microscopic examination of placentas from mothers with type 1 diabetes did not differ significantly from those of controls [41]. Specifically, measures that could be expected to have an effect on substrate diffusion (villous surface area and membrane thickness and capillary surface area, placental morphometric diffusing capacity) were very similar in both groups. This was despite evident differences in foetal insulin and leptin concentrations. Furthermore, foetal IGF-1 concentrations but not insulin or leptin, or maternal diabetes or HbA1c, were associated with placental substructure [41]. On the other hand, when compared with those of mothers with type 1 diabetes, placentas from women with type 2 diabetes show more frequent maternal decidual vasculopathy and placental insufficiency, despite better glycaemic control at the beginning of the pregnancy [42].

Placental gene expression differ in women with gestational diabetes and those with normal glucose tolerance [43,44,45]. Although results vary among studies, genes involved in substrate metabolism, immunity-inflammation and transport have been reported to be differentially expressed [43,44,45]. A small study assessing placental gene expression (using a metabolic gene array) in women with type 1 diabetes, obese women with gestational diabetes and women without diabetes, showed differential regulation of lipid-metabolism related genes in the women with gestational diabetes [46]. More recent studies have tried to discriminate the effects on gene expression caused by gestational diabetes itself and those associated with obesity, revealing upregulation in gestational diabetes of genes involved in RNA processing and splicing [47], as well as divergent expression of genes such as *mTOR* and *AMPK* [48]. Placental miRNA expression in gestational and pre-gestational diabetes is reviewed in Section 2.3 and Section 2.4.

### 2.2. MiRNAs and the β-Cell

Pregnancy is associated with maternal β-cell expansion, to account for the increased insulin needs associated with insulin resistance (reviewed in Reference [49]). Studies performed in pregnant rats support a role of miRNA, especially miR-338-3p, which reduces its expression at the time of maximal β-cell expansion. Furthermore, inhibition with a specific anti-miR leads to increased β-cell proliferation [50]. The opposite is true for miR-451, which is upregulated in the islets of pregnant rats. In vitro over-expression of miR-451 and blockade of miR-338-3p protect β-cells from cytokine- and palmitate-induced apoptosis in rat islets [50].

A large suite of miRNAs has been implicated in pancreas development, the most important being miR-15a/b, miR-124a, miR-7, miR-376 and miR-375 (reviewed in Reference [51]). The miRNA signature of the human developing pancreas (gestational weeks 10 to 22) was first described by Rosero et al., determining specific groups of miRNAs that change their expression throughout development [52]. The authors identified 212 miRNAs, of which 39 showed expression changes with advancing gestation and correlated to target mRNAs and found that most miRNAs correlate with multiple targets, showing a two-way level of combinatorial control regulating gene expression [52]. The study did not distinguish exocrine from endocrine pancreas, so β-cell specific miRNAs could not be ascertained. Other studies have identified miR-375, miR-376, miR-9 and miR-7 as islet-specific [53]. Indeed, the expression of miR-375 and miR-7 increased with increasing gestational age and increasing insulin production and their expression partially co-localised with insulin-positive cells, although they did not seem to be β-cell specific [53]. MiR-375 is also involved in the regulation of insulin secretion, as well as cellular growth and proliferation and α and β-cell phenotype maintenance (reviewed in Reference [54]). Regardless of their implication in pancreas development, other miRNAs have been involved in β-cell function, the most consistent probably being miR-29a-3p, miR-25-3p [54], miR-21-5p, miR-24-3p and miR-148a-3p (reviewed in References [54,55]).

Maternal under and over-nutrition have been associated with increased risk of β-cell dysfunction and type 2 diabetes in the offspring [49]. Low-protein diet in pregnant rats has an impact on foetal pancreatic miRNA expression, including but not limited to, upregulation of miR-375 [56]. The latter was associated with reduced cell proliferation and insulin secretion [56].

The study of miRNA in pregnancy and its associated complications, such as preeclampsia, foetal growth restriction and preterm labour, is a young field [36] and so is the study of miRNA in the diabetic mother.

### 2.3. Gestational Diabetes and miRNAs

Both the physiology [3] and the epigenetics of gestational diabetes have recently been reviewed [57,58,59]. More than twenty human studies have been published describing the association of gestational diabetes with certain miRNAs, most of them in the last couple of years. Their heterogeneity, reflected by their diverse features, is displayed in Table 2 [60,61,62,63,64,65,66,67,68,69,70,71,72,73,74,75,76,77,78,79,80,81,82]. Their sample sizes range from 6 individuals per group to about 200 subjects and gestational age, when described, ranges between 7 weeks and term (37–39 weeks). The majority of the studies have used maternal peripheral blood samples, either serum, plasma or whole blood [61,62,63,65,67,70,71,72,74,78,79,81], whereas others have used maternal tissues, such as omental fat [73]. Others have assessed placenta [60,68,69,76,79,80,82] or umbilical vein endothelial cell specimens [64,77], obtained after vaginal delivery or caesarean section. To the best of our knowledge, only one study has assessed miRNA expression in adult offspring of mothers with gestational diabetes [66]. Regarding the study approach, thirteen have performed unbiased, massive miRNA profiling, either by means of an array [63,67,68,70,72,73,75,77,80,81] or by next generation sequencing methods [62,74,80] whereas the rest have assessed pre-selected miRNAs. Internal control selection for miRNA expression normalization is not uniform, either, though the most frequently used control is small nuclear RNA (snRNA) U6. Other studies do not specify [79], or do not seem to use endogenous controls for normalization [67,70,75,81]. The diagnostic criteria of gestational diabetes also vary from one study to the next, although most of those who specify them, use either the two-step approach with a 3-h, 100 g oral glucose tolerance test [5] or the one-step, 2-h, 75 g approach proposed by the International Association of Diabetes in Pregnancy Group [8]. Given all these differences among the studies, comparison of their results is far from straight-forward. For comprehension and readability’s sake, studies have been classified and will be discussed based on sample selection and main objective of the study (biomarker assessment vs. mechanistic explanation).

#### 2.3.1. Studies in Maternal Blood

The most obvious clinical application of the study of miRNAs in maternal blood is the identification of biomarkers to predict gestational diabetes or its complications.

Zhao et al. performed a low density miRNA array, followed by quantitative polymerase chain reaction (qPCR) validation, in women in their early pregnancy (16–19 weeks), some of whom later (week 24–28) developed gestational diabetes. In the latter, the expression levels of three miRNAs (miR-132, miR-29a and miR-222) were significantly decreased when compared to controls at similar gestational weeks. Replication in two other external sample sets confirmed miR-29a and miR-222 as useful biomarkers. In vitro, miR-29a inhibition increased Insulin-induced gene 1 (Insig1) expression in HepG2 cell lines [81]. In a more recent (pilot discovery) study with a similar temporal design, miRNA profiling through massive sequencing with further validation by qPCR suggested other predictors of gestational diabetes to be assessed: miR-16-5p, miR-17-5p, miR-19a-3p, miR-19b-3p and miR-20a-5p [62]. Based on these results, Cao et al. performed serial sampling during pregnancy in women who developed gestational diabetes and in women who did not. Their results support miR-16-5p, miR-17-5p and miR-20a-5p as the best predictors of gestational diabetes, with areas under the receiver operating characteristics (ROC) curve of 0.92, 0.88 and 0.74, respectively [61]. MiR-20a-5p was also confirmed as the best biomarker in a more recent evaluation of selected candidates, although its predictive value was low and added little value to classical risk factors of gestational diabetes [70]. Another study, performed in 36 women who developed gestational diabetes and 80 women who did not, assessed 10 plasma miRNAs in early-mid pregnancy, selected from previous studies on pregnancy outcomes. Overall, gestational-age adjusted expression of miR-155-5p and miR-21-3p were positively associated with gestational diabetes. However, results differed in lean and obese women and depending on offspring sex [78].

Sebastiani et al. performed an unbiased array of 384 miRNAs, of which two were validated by qPCR. MiR-330-3p was upregulated in gestational diabetes and correlated with pregnancy outcomes, that is, women who needed a caesarean section showed higher expression of this miRNA [72].

Other studies assessing miRNA expression in the blood of women with gestational diabetes are more mechanistic and not quite as applicable to clinical practice.

A study assessing twelve miRNAs, related to neural development, in maternal serum, showed changes in expression during pregnancy and significant differences in expression between women without diabetes and women with gestational diabetes. Indeed, the expression of miR-125-5p increased in the second and third trimesters in normal pregnancy and a relatively increased expression was found in the first trimester in women with gestational diabetes. In the second trimester, expression in women with gestational diabetes was 10-fold lower than in controls. The authors hypothesise that adverse intrauterine conditions, such as gestational diabetes, alter cell proliferation and neurogenesis in the first trimester and that this can be detected in maternal serum [67].

In a group of women with gestational diabetes and age- and BMI-matched controls, Stirm et al. assessed miRNA profiles in maternal whole blood cells. They identified 29 miRNAs which were upregulated in maternal blood, after adjusting for pregnancy weight-gain and gestational age. Of the 29 miRNAs, only miR-340 could be validated by qPCR. Lymphocytes from non-pregnant women were treated in vitro with insulin and with glucose, showing an increase and reduction in miR-340 expression, respectively. Cord blood was collected from eight offspring born from glucose tolerant women and eight from women with gestational diabetes (adjusted for foetal sex and length) but no differences were found between the two groups in the expression of miR-340. They concluded that their study provides evidence for insulin-induced miRNA-dependent programming of maternal white blood cells [74]. In addition, associations between BMI and miRNA expression were also explored, with correction for gestational diabetes, maternal age, pregnancy week and maternal weight gain. Four miRNAs showed a positive association with maternal BMI: miR-4473, miR-199a-5p, miR-339-5p and miR-3653-5p [74].

#### 2.3.2. Studies in Placenta

Placental studies have mostly assessed preselected, single miRNAs, such as miR-143, which was down-regulated in insulin-treated but not in diet-treated gestational diabetes and was associated with an altered metabolic switch between oxidative phosphorylation and aerobic glycolysis [69]. Another example is miR-518d, which is up-regulated in gestational diabetes. PPAR-α is a predicted and validated target, with inverse placental protein expression [82]. In a larger study, miR-98, which was also upregulated in the placenta of women with gestational diabetes, showed an association with increased global DNA methylation, highlighting the complex relationship between different epigenetic modifications [60].

Finally, circular RNAs (circRNAs), other non-coding RNAs which have been described to regulate miRNA expression, have recently been studied in gestational diabetes [80]. Of the almost 50,000 circRNAs detected in placental villi by next generation sequencing, 227 were significantly upregulated and 255, down-regulated in the samples of women with gestational diabetes. Biological pathway prediction showed enrichment of processes involved in glucose and lipid metabolism [80].

Other tissues have also been studied, such as maternal omental fat. miR-222, 1 of 17 identified differentially expressed miRNAs, was found to be significantly up-regulated in the omental fat of women with gestational diabetes and its expression was correlated with serum oestradiol concentrations. Indeed, oestrogen receptor (ER)-α protein and insulin-sensitive membrane transporter glucose transporter 4 (GLUT4) protein, targets of miR-222, were distinctly reduced [73].

Taking the heterogeneous nature of the studies into consideration and the overall lack of replication in their results, very few conclusions can be drawn on their applications:

- Some miRNAs could potentially be used as predictors of gestational diabetes and perinatal outcomes.
- The most promising markers of gestational diabetes are: miR-29a, miR-222, miR-16-5p, miR-17-5p and miR-20a-5p.
- Some studies propose a role for miRNAs in the pathogenesis of gestational diabetes and its complications but no conclusive information is available yet. Other non-coding RNAs may also play a role in the pathogenesis and consequences of gestational diabetes.

Potential causes for the lack of replication in the studies include:

- Sample source: although some overlap has been described in the results obtained in placental and peripheral blood samples, this is not the rule. Indeed, regarding blood samples, some studies use serum or plasma, whereas others use lysed, whole blood or leukocytes (see Table 2).
- miRNA expression varies with gestational age. Thus, control groups need to be matched to the study group by this variable.
- Other factors, such as mode of delivery, offspring sex and BMI could also add to the risk of bias.
- The diagnostic criteria used to define gestational diabetes also vary among studies, although this is probably a minor, if any, source of bias. All definitions have in common mild hyperglycaemia diagnosed, for the first time, during pregnancy.
- As applies to miRNA studies in other contexts, isolation methods and choice of valid, endogenous controls for expression normalization can be crucial. Furthermore, the choice of optimal endogenous controls for one tissue might not be valid for other sample sources [83,84].

Some recommendations for future studies follow:

- Gestational age should be reported and matched between the study and control groups.
- When placenta samples are obtained, given the mixed nature of this organ, the procedure needs to be standardized to minimize bias. Furthermore, the mode of delivery needs to be reported, as well as offspring sex
- When used as biomarkers of gestational diabetes or its outcomes, the performance of miRNAs should be compared with that of classical, easy-to-assess risk factors.
- The criteria for selecting a certain miRNA or group of miRNAs for assessment or validation should be clear, as well as the choice for endogenous controls.

### 2.4. Pre-Gestational Diabetes and miRNAs

In contrast to what happens in gestational diabetes, women with established type 1 or type 2 diabetes who become pregnant may already have changes in body tissues (like atherosclerosis, presence of advanced glycation end products) and have altered metabolic functions (oxidative stress, mitochondrial dysfunction, etc.) that can interfere with conception and pregnancy. Indeed, hyperglycaemia may have an especially relevant impact in early pregnancy of diabetic mothers, where critical development of the embryo and foetal membranes, as well as extensive epigenetic programming, take place.

To date, there are no broad miRNA expression profiling studies published of human offspring exposed to prenatal hyperglycaemia, although studies are now starting to emerge.

In a study assessing muscular biopsies in offspring of women with type 1 diabetes and gestational diabetes (see Table 1), miR-15a and miR-15b, selected due to their potential role in insulin secretion and resistance, were found to be overexpressed in both study groups, when compared to offspring of mothers with normal glucose tolerance. Furthermore, the expressions of both miRNAs were correlated with measures of glycaemic control in the study subjects and with maternal 2-h post oral glucose tolerance concentrations during pregnancy in the offspring of the mothers with gestational diabetes [66].

Our group performed a study where samples of the maternal and foetal sides of the placenta were obtained from women with type 1 and type 2 diabetes, women whose partner had type 1 diabetes and healthy controls matched for age and gestational age. Ten pools of 8–10 samples of maternal and foetal placenta were analysed and massive miRNA sequencing was performed [85]. Eight miRNAs were validated by qPCR in the 96 samples composing the pools and data mining was used to identify potential biomarkers, specific for type 1 and for type 2 diabetes. Six classifiers were found for type 1 and 4 for type 2 diabetes, all composed of 2–3 miRNAs. MiR-125-5p and miR-20a-5p were present in several classifiers, both for type 1 and type 2 diabetes, suggesting that they are related to maternal hyperglycaemia [86]. This is supported by their previous identification as biomarkers of gestational diabetes [61,62,67]. Replication studies and association of miRNA expression with pregnancy outcomes are currently ongoing [86].

#### miRNA and Diabetic Embryopathy

The most studied mechanism for miRNAs in pre-gestational diabetes is the potential embryopathy of the offspring, affecting several organs, especially the neural tube and the heart. Existing evidence is based on mouse and in vitro studies (see Table 3) [86,87,88,89,90,91,92,93,94,95].

Pre-gestational diabetes alters gene expression, which can lead to neural tube defects and miRNAs have been shown to regulate genes involved in brain development and maturation. Several studies have described hyperglycaemia-induced epigenetic changes in neural stem cells which alter gene expression and cell fate during brain development (reviewed in Reference [96]). miRNA profiling in neural stem cells suggested that pre-gestational diabetes alters genes involved in neural tube formation via miRNA regulation. The miR-30 family was found to be upregulated in neural stem cells obtained from embryos of diabetic dams, compared to those of controls. miR-30b was found to be upregulated and its target gene *Sirtuin 1* was down regulated, leading to decreased expression of the Sirtuin 1 protein, which was associated with altered neuron/glia ratio [90]. Furthermore, decreased expression of four miRNAs targeting *Dcx* (Doublecortin) and *Pafah1b1* (Platelet activating factor acetyl hydrolase, isoform 1b, subunit 1)—involved in neurogenesis and neuronal migration—was found in neural stem cells obtained from embryos of diabetic mice. In addition, these epigenetic changes persisted, even when the cells were cultured in normoglycaemic conditions [92]. Another study in murine neuroepithelial cells described that miR-192-2 can mediate the teratogenicity of hyperglycaemia leading to neural tube defects. It acts as a negative autophagy regulator, diminishing neural tube defects by targeting the peroxisome proliferator-activated receptor-γ-coactivator (*PGC-1α*) gene, a positive regulator of mitochondrial function, which is disturbed in pre-gestational diabetes [93]. miR-17 repression mediated the pro-apoptotic effect of high glucose, removing thioredoxin inhibition on Apoptosis Signal-regulating kinase 1, which triggered neural stem cell apoptosis of the developing neuroepithelium and led to neural tube defects [88].

Congenital heart defects were also studied in C57BL/6 mice with streptozotocin-induced diabetes. Significant changes in blood exosomal miRNAs, involved in cardiac development, was observed. Furthermore, the study showed that labelled exosomes could cross the placental barrier and infiltrate embryonic tissues, including the heart, during embryonic development. The injection of these exosomes strikingly increased the risk of congenital heart defects in non-diabetic mice [91]. A total of 149 miRNAs, whose expression was altered by pre-gestational diabetes, were mapped in the developing heart of mice offspring and their target genes were associated with cardiac development-related pathways (STAT3 and IGF-1 and several transcription factors). Furthermore, suppression of oxidative stress, by an antioxidant enzyme, reversed the altered expression of the miRNAs associated to pre-gestational diabetes [87].

Another study assessing early effects of oxidative stress in pre-gestational diabetes described a role for miR-27a. At 8.5 days after conception, embryos of diabetic dams showed a decrease in the mRNA and protein levels of nuclear factor erythroid 2-related factor 2, a master regulator of the cellular antioxidant system. This was similarly proven in cultured neural stem cells in response to high glucose in a dose and time dependent manner, which resulted in increased miR-27a expression, explaining the suppression of its target nuclear factor erythroid 2-related factor 2 and its responsive antioxidant enzymes, associated with diabetic embryopathy [95]. In a study of female mice with streptozotocin-induced diabetes, miRNA expression profiling was performed in the embryos, at different gestational days, with next generation sequencing. On embryonic day 8.5, the abundance of expressed miRNAs was similar in the offspring of diabetic and of normoglycaemic dams. One day later, 3 miRNAs (miR-505-5p, miR-770-5p and miR-1a-1-5p) were differentially expressed but their putative target genes were underrepresented in a database of genes associated with cardiovascular or neural malformations [94].

Beside the embryo, maternal diabetes during pregnancy is also associated with abnormal placental mitochondrial function and oxidative stress, which can lead to altered foetal development and reduced long term health. PGC-1α, a master regulator of mitochondrial biogenesis and energy metabolism, was found to be decreased in a human placental trophoblastic cell line exposed to high glucose. The latter increased the expression of miR-130b-3p and its exosomal secretion and its transfection to the cells mimicked the downregulation of *PGC-1α* expression. The authors suggest that the reduction in PGC-1α could explain some of the metabolic features of diabetes and also the increased risk of diabetes in children born to women with diabetes in pregnancy [89].

### 2.5. miRNA and Macrosomia

Foetal macrosomia is the most common complication of gestational and pre-gestational diabetes and is associated with an increased risk of neonatal hypoglycaemia and respiratory distress, as well as life-long risk of obesity and diabetes [3,97,98]. Despite the improvement in the diagnosis and management of hyperglycaemia, the prevalence of diabetes macrosomia is increasing worldwide, parallel to the growing prevalence of diabetes and obesity [97]. With respect to non-diabetic macrosomia, there is still no clear consensus regarding its prediction and management [99]. Thus, early biomarkers of macrosomia as well as therapeutic options are necessary.

Foetal growth requires a complex interaction between the mother, the placenta and the foetus with environmental factors [100]. Factors known to be involved in the development of macrosomia are both genetic and environmental, such as hyperglycaemia, hyperlipidaemia, obesity and maternal age [101]. However, molecular mechanisms that contribute to macrosomia are still poorly understood and there is growing evidence that the intrauterine environment can have an impact on this outcome, influencing gene expression through epigenetic mechanisms [102,103]. In line with this, recent studies have demonstrated that foetal growth can be regulated by non-coding RNAs [103,104,105,106,107]. To better understand the role of the non-coding RNAs in macrosomia, it is necessary to consider this condition both in the context of the diabetic pregnancy and in the non-diabetic pregnancy.

Different approaches have been used to evaluate non-coding RNAs in macrosomia. Placenta, peripheral blood from mothers at different gestational stages, umbilical cord blood and neonatal blood have been evaluated to establish both reference and altered expressions in a vast number of non-coding RNAs [68,99,103,106,108,109,110] and miRNAs represent the main transcripts in most of the studies.

One of the main aims of these studies has been to develop biomarkers for early diagnosis of macrosomia [99,106]. Because miRNAs are present in all body tissues, blood and other fluids and are stable and protected against RNase activity, their potential value as diagnostic and prognostic biomarkers is evident, in addition to their potential therapeutic role [99,103,106,108,110].

Hu and collaborators screened the role of miRNAs as potential early-diagnostic biomarkers for macrosomia, finding that the low expression miR-376a can serve for diabetic macrosomia [99]. MiR-376a has been implicated in different types of cancer and is considered an important predictive biomarker for obesity and type 2 diabetes [111,112].

Ge and collaborators analysed the expression of an array of miRNAs at 18–28 weeks of gestational age in macrosomic and healthy pregnancies [108]. Of the 143 differentially expressed miRNAs, 12 were more thoroughly evaluated by qPCR. miR-30a-3p, miR-125a-5p, miR-523-3p and miR-661 were upregulated, whereas miR-18a-5p, miR-141-3p, miR-143-3p, miR-181a-5p, miR-200c-3p, miR-221-3p and miR-451a, were downregulated. Most have been associated to obesity or diabetes (miR-16-5p, miR-125a-5p, miR-143-3p, miR-221-3p) [113,114]. In general, they target specific immunological, cell signalling and cycle processes intimately related with gestation [108]. miR-523-3p, miR-141-3p and miR-200c-3p showed good diagnostic efficiency for macrosomia and the two latter, also distinguished macrosomia from other pregnancy complications, like preeclampsia [108]. Four clusters were also evaluated (miR-17-92, miR-27, miR-451 and miR-141) that were differentially expressed in macrosomic pregnancies, with high sensitivity and specificity [108].

The expression of circulating miRNAs varied at the different stages of gestation, interacting with foetal growth restriction [115,116]. Jiang et al. studied the variability in the expression of miRNAs in maternal plasma at 16–20 weeks of gestational age and 1 week before delivery, in women with macrosomic and healthy gestations [95]. One week before delivery, 32 miRNAs differed in their expressions between macrosomic pregnancies and controls and miR-21 was selected for its potential validity. MiR-21 has been related to different processes and diseases like type 2 diabetes, obesity and foetal growth [117,118,119]. Indeed, miR-21 regulates adipocyte differentiation and proliferation and its expression is positively correlated with body mass index [119,120].

Jiang and colleagues did not find differences in the expression of miR-21 at the early second trimester but observed lower serum expression in the third trimester in macrosomic pregnancies. They also demonstrated its validity as a diagnostic biomarker for this condition at this stage [103], although its usefulness at the end of pregnancy is limited. These results are not fully consistent with other previously obtained in placenta from macrosomic neonates, where miR-21 was overexpressed [105].

However, it is plausible that its upregulation in the placenta may produce macrosomia, through the stimulation of cell differentiation and proliferation [103]. miR-21 targets several pathways like the mitogen-activated protein kinase (MAPK) and the Janus kinase (JAK)-signal transducer and activators of transcription (STAT) (JAK-STAT) signalling pathways [103]. The MAPK signalling pathway is crucial for the cellular proliferation, growth and apoptosis [107]. The JAK-STAT signalling pathway regulates body growth and lipid and glucose metabolism [121]. In contrast, the suppressors of cytokine signalling (SOCS) protein family is involved in the negative regulation of JAK-STAT-dependent activities [122,123,124]. SOCS-2 plays a key role in body growth and development, lipid and glucose metabolism [125,126]. Recent characterisation of the *Socs2*^−/−^ mouse supports a role for this protein in macrosomia and gestational diabetes [127].

The role of aberrant placental miRNA expression in the regulation of foetal growth and gestational complications have been demonstrated [118]. The low expression of miR-143 in placenta has been associated with diabetes macrosomia and its upregulation is associated with protection from it [107]. miR-143 expression regulates adipocyte differentiation and growth [128,129]. It is also a fundamental regulator of several cellular activities, targeting other multiple mRNAs for cell proliferation or apoptosis [99]. In line with its expression in placenta, miR-143 is also downregulated in maternal blood in macrosomic pregnancies [130].

The target genes affected by miR-143, similar to miR-21, include the *MAPK*, in this case through a counter regulatory action which induces cell proliferation and growth [131].

Interestingly, this miRNA interacts with miR-21 determining the risk for diabetic macrosomia [107]. This risk is enhanced with an opposed expression of both RNAs, with low miR-143, simultaneous to a high expression of miR-21. On the contrary, simultaneous up- or down-regulation of both miRNAs is not associated with higher risks of macrosomia [107].

Placental miRNA expression in macrosomia has also been studied based on miRNA clusters. One of these clusters is associated to the EGFR/PI3K/Akt (epidermal growth factor receptor/phosphoinositide 3-Kinase/protein kinase B) signalling cascade [68]. This cluster is comprised by the upregulation of miR-508-3p and the down-regulation of miR-9, miR-27a, miR-30d, miR-33a, miR-92a, miR-137, miR-362-5p and miR-502-5p. Their overall interaction enhances epidermal growth factor receptor signalling, via the EGFR/PI3K/Akt cascade, with consequent foetal overgrowth [68]. MiR-508-3p is upregulated during gestational diabetes and seems to directly target and suppress *PIKfyve* (FYVE-containing phosphoinositide 3-phosphate (PI3P) 5 kinase) that phosphorylates phosphatidylinositol (PtdIns) 3P to PtdIns (3, 5) P2, a key inhibitor of EGFR [109].

Placental expression of the cluster miR-17-92, formed by miR-17, miR-18a, miR-19a, miR-20a, miR-19b and miR-92a, has been associated to diabetic foetal macrosomia, promoting cell proliferation and reducing apoptosis [132]. Some of its members were previously identified to be deregulated in the maternal plasma of macrosomic pregnancies [108]. Moreover, the validity of this cluster as a diagnostic tool for macrosomia using maternal serum, has also been demonstrated [106].

Analysing dried blood spots of newborn screening cards, Rodil-García and colleagues recently demonstrated, that the expressions of miR-33b, miR-375 and miR-454-3p, are also upregulated in macrosomia. However, miR-454-3p, was also upregulated in low-birth weight babies [110]. MiR-33b and miR-375 are related to the metabolism of glucose and lipids and insulin signalling or secretion [49,50,133], whereas miR-454-3p has a role in inflammatory response and postnatal brain development [134,135]. Specifically, miR-375 is related to pancreatic islet development and adipogenesis and its expression is regulated by glucose [49,50].

In summary, the complex mechanisms that interact in macrosomia are still not fully understood. The interplay of an important number of miRNAs has been mapped, but further research is required to establish the diagnostic and therapeutic validity of these potential biomarkers.

### 2.6. Paternal Effects on Offspring and miRNAs

Developmental Origins of Health and Disease are not necessarily limited to maternal-dependent effects but fathers also have a proven role in the future disease risk of their offspring [136], reviewed in Reference [137]. Paternal obesity has been associated with impaired sperm quality and quantity and subsequent subfertility [138,139]. In couples receiving assisted reproduction, paternal obesity was associated with decreased blastocyst development and lower clinical pregnancy and live birth rates as a result of the procedure [140]. In addition, a recent meta-analysis revealed that deficient peri-conceptional paternal nutrition has a similar effect on offspring weight than maternal nutrition [20] and epigenetic mechanisms have been proposed as an explanation [141], although little evidence is yet available for miRNAs.

Male mice that were fed a moderately-high (21%) fat diet for ten weeks, developed obesity but not diabetes, although there was a trend towards higher glucose concentrations [142]. Their offspring, especially the females, also developed obesity, despite receiving a normal diet. The sperm of their fathers showed global DNA de-methylation, as well as changes in the expression of 4 miRNAs. miR-133b-3p, miR-196a-5p and miR-205-5p were up-regulated, whereas miR-340-5p was down-regulated. Ingenuity pathway analysis limited to the target genes with differential expression in the high-fat diet mice, showed enrichment in networks involved in nitric oxide and ROS pathways, Sertoli cell junction signalling, EIF2 signalling, NF-κβ signalling and inflammatory response and lipid and carbohydrate metabolism [139]. The authors postulated that miRNAs are involved in transgenerational transmission of acquired paternal features [139]. On the other hand, in the offspring of (male) mice fed a low-protein diet, moderate upregulation was found in the hepatic expression of some miRNAs (miR-21, let-7, miR-199, miR-98) and down-regulation in another (miR-210). However, target mRNAs did not show consistent expression changes [142], suggesting that miRNA regulation might not be the main mechanism involved in the metabolic response of the offspring.

The epigenetic effects of paternal diabetes on the offspring have received less attention, so far. Type 1 diabetes has been associated with reduced fertility in men, albeit to a smaller extent than in women [143,144]. The association between sperm quality and diabetes is controversial. Although light microscopy analysis was normal according to a study, nuclear DNA fragmentation and mitochondrial DNA deletions were more frequent in men with diabetes [145] and mitochondrial membrane potential was reduced [146]. In the latter study, no correlation was found between sperm abnormalities and glycaemic control or diabetes duration [146].

In offspring of prediabetic male mice, impaired glucose tolerance and reduced insulin sensitivity were observed. Furthermore, overall gene methylation was increased in their pancreatic islets and gene expression was altered in 402 genes. Indeed, different methylation patterns were also found in the sperm of the prediabetic fathers, which showed high concordance with that of pancreatic islets in the offspring [147]. In an elegant study performed in mice which developed obesity and glucose intolerance induced by a high-fat (60%) diet, small, non-coding RNA was identified as the vector of transgenerational transmission of metabolic disorders. However, miRNA expression was similar in the sperm of males fed high-fat and normal diets but a set of 30–34 nucleotide RNAs, derived from tRNA, was responsible for the inheritance of the diet-induced metabolic disorders [148].

To summarise, paternal nutrition and metabolic status have a proven effect on the offspring and epigenetic effects may play a role. Although there is limited evidence of a role of miRNAs in this context, other short, non-coding RNAs may be involved in transgenerational transmission of metabolic disorders. Little is known about the specific role of non-coding RNA in paternal transmission of diabetes-related complications.

## 3. Conclusions

Although still in its infancy, miRNA research in diabetic pregnancy has exploded in the last few years. Gestation is associated with β-cell expansion, which might be regulated, at least to a certain extent, by miRNAs. Early rat studies suggest a role for miR-451 and miR-338-3p in this expansion. The miRNA profile of the embryonic pancreas has been characterized and some miRNAs seem to be islet-specific (miR-375, miR-376, miR-9 and miR-7), although β-cell specific miRNAs are still to be identified. Regarding gestational diabetes, the most promising research so far is probably the identification of biomarkers of the disease and its outcomes: miR-29a, miR-222, miR-16-5p, miR-17-5p and miR-20a-5p are the best validated. In pre-gestational diabetes, most miRNA research has focused on diabetic embryopathy, especially neural and cardiac malformations, which have been assessed in mice and in in vitro studies, although results are not consistent yet. In diabetic macrosomia, the interplay of an important number of miRNAs, often acting in clusters, has been evaluated but further research is still needed. Little is known about the specific role of non-coding RNA in paternal transmission of diabetes-related complications.

For future research in miRNAs during pregnancy, several aspects should be borne in mind when performing case-control studies: miRNA expression varies with gestational age, type of sample studied, offspring sex and maybe also with mode of delivery. Thus, these variables should be balanced between the study and control groups.

## Figures and Tables

**Table 1:** Diagnostic criteria for gestational diabetes based on plasma glucose concentrations after an oral glucose tolerance test.

	After 100 g Glucose [6]	After 100 g Glucose [7]	After 75 g Glucose [8,9]
Fasting	95/5.3	105/5.8	92/5.1
1 h	180/10.0	190/10.6	180/10.0
2 h	155/8.6	165/9.2	153/8.5
3 h	140/7.8	145/8.0	--

Glucose concentrations are expressed in mg/dL and mmol/L. Meeting two or more thresholds is usually required for diagnosis, although a single value is sometimes used (reviewed in Reference [5]).

**Table 2:** Studies assessing the role of miRNAs in gestational diabetes (GD) and its consequences.

Author Year [Reference]	miRNA	Methods/Control	Diagnostic Criteria	GA (wk)	N (GD/C)	Tissue	Results	Comments
**Maternal blood**								
Zhao 2011 [81]	miRNA profiling miR-132 miR-29a miR-222	Array (Applied Biosystems)+qPCR/cel-miR-36	At 24–28 weeks. Two-step. 75 g 3 h OGTT	16–19	36/36	Serum	Reduced in the women developing GD	Profiling in two pools of 24 samples. 10 miRNA validated. miR-222, miR-29a validated in 2 external samples (16/group in each centre)
Zhu 2015 [62]	miRNA profiling miR-16-5p miR-17-5p miR-19a-3p miR-19b-3p miR-20a-5p	Massive sequencing profiling+qPCR/miR-221	Two-step. 75 g, 3 h OGTT	16–19	10/10 (one pool each)	Plasma	Upregulated in the women developing GD	Pilot discovery study. The 5 mentioned miRNAs, validated with q-RT-PCR also in the pools, apparently
Cao 2017 [61]	miR-16-5p miR-17-5p miR-20a-5p miR-19a-3p miR-19b-3p	qRT-PCR/U6	Three-hour 75 g OGTT	Serial sampling until week 24–28	85/72	Plasma	miR-16-5p, miR-17-5p, miR-20a-5p up-regulated in GD at diagnosis miR-16-5p and miR-17-5p in earlier pregnancy. AUC ROC 0.92 for 16-5p	Selection based on [56]
Wander 2017 [78]	miR-126-3p miR-155-5p miR-21-3p miR-146b-5p miR-210-3p miR-222-3p miR-223-3p miR-517-5p miR-518a-3p miR-29a-3p	qPCR/miR-423-3p	Two-step diagnosis 100 g OGTT [6]	7–22	36/80	Plasma	miR-155-5p and miR-21-3p up-regulated in GD Differential regulation according to maternal obesity and offspring sex. Analysis adjusted for gestational age (logistic regression)	Ten candidate miRNA selected based on previous association with pregnancy complications
Pheiffer 2018 [70]	miR-16-5p miR-17-5p miR-19a-3p miR-19b-3p miR-20a-5p miR-29a-3p miR-132-3p miR-222-3p	qPCR array (Qiagen)/Cel-miR-39	Two-hour 75 g OGTT at 24–28 weeks of pregnancy [8]	13–31	28/53	Serum	miR-20a-5p and miR-222-3p down-regulated in GD	miRNAs selected for previous association with GD. Only 20a-5p significant predictor of GD in multivariate logistic regression analysis
Tagoma 2018 [75]	Array of 84 miRNA miR-195-5p	miRNA array+qPCR/Cel-miR-39	Two-hour OGTT with 75 g glucose during the second trimester of pregnancy [8]	23–31	13/9	Plasma	miR-195 upregulated in GD	Higher gestational age in GD. Of the 84 miRNA array, 15 were upregulated in GD. Top 3 were validated with qRT-PCR. miR195-5p has targets in lipid metabolism
Sebastiani 2017 [72]	Array of 384 miRNAs miR-330-3p	TaqMan miRNA Human Array Panel A platform (Life-technologies)+qPCR/miR-320 and miR-374a	Two-hour 75 g OGTT at 16–19 weeks or 24–28 weeks of pregnancy [8]	24–33	25/14$	Plasma	miR-330-3p upregulated in GD	Bimodal expression observed in GD. Low expression associated with less caesarean sections
Collares 2013 [63]	miRNA profiling	Array (Agilent)/Median expression, quantile normalization	GD vs. non-pregnant DM1 and DM2	28–37	6/7/7	Blood (PBMC)	The authors conclude that miRNA profiles distinguished types of diabetes	Non-pregnant DM1 and DM2 differed from GD in age and sex distribution, as well as pregnancy state
He 2017 [65]	miR-494	RT-qPCR/U6	NS	NS	20/20	Peripheral blood	Down-regulated in GD Overexpression of miR-494 enhanced insulin secretion and increased total insulin content, induced cell proliferation and inhibited cell apoptosis in INS1 cells	From pre-existing database in Chinese women with differential expression in GD. MiR-494 chosen because of relation with apoptosis in other tissues
Lamadrid 2018 [67]	miR-125b-5p and 11 other miRNA	RT-qPCR array/Cel-miR-39-3p	American diabetes association 2016 two-step protocol (any trimester)	Sampling in 3 trimesters	14/27	Serum	miR-183-5p, miR-200-3p, miR-125b-5p, miR-1290 higher in GD in first trimester	miRNAs selected because of previous association with neural development
Rahimi 2014 [71]	Drosha, Dicer, DGCR8 mRNA	qPCR/RPL38 mRNA	NS	Average 32–33 (SD2.7)	20/20	Whole blood-lysis	Drosha and Dicer Upregulated and DGCR8 down-regulated in GD	Components of the miRNA machinery are altered in GD
Stirm 2018 [74]	miR-340	miRNA profiling (massive RNA sequencing-Illumina HiSeq. 2500 platform)+qPCR/RNU6B	IADPSG recommendations [8]	24–32	30/30	Whole blood cells from mothers and offspring, lymphocytes	Upregulated in GD and up-regulated by glucose, down by insulin, in vitro (lymphocytes)	Screening in 8/8, validation in 30/30 and 8/8 offspring. 29 miRNA upregulated in GD, one validated by qRT-PCR.
Xu 2017 [79]	miRNA profiling Validation of miR-503	Array (Agilent)+qPCR/NS	NS	NS	3/3 25/25	Placenta Maternal peripheral blood	Upregulated in GD. In vitro inhibition of miR-503 increases insulin content and secretion in INS1 cells	One of the 28 upregulated miRNAs in an array was selected. Array in placentas, validation in blood
**Placenta**								
Cao 2016 [60]	miR-98	qRT-PCR/U6 snRNA	NS	39+/−1	193/202	Placenta	Upregulated in GD, increases global methylation	Single miRNA
Li 2015 [68]	miR-508-3p miR-27a miR-9 miR-137 miR-92a miR-33a miR-30d miR-362-5p miR-502-5p	Array (Agilent), qPCR/U6 snRNA	Fasting glucose >5.1 mmol/L	Term	15/15 *	Placenta	29 differently expressed miRNA in the array, 9 replicated by qPCR. miR-508-3p upregulated and the rest, down-regulated	In silico prediction shows EGFR/PI3K/Akt pathway involvement, which plays a role in foetal growth. EGFR/PI3K/Akt upregulated in GD placentas
Muralimanoharan 2016 [69]	mir-143	qPCR/U18 RNA	NS. Include A1 and A2	Term (38–39), caesarean section only in GD	12/6	Placenta (trophoblasts)	50% reduction in A2 but not A1 GD	Selected for previous association with metabolic switch between glycolysis and oxidation. Overexpression of miR-143 reduces aerobic glycolysis and rescues mitochondrial complexes in trophoblast cells
Tan 2016 [76]	miR-95 miR-548 miR-1246	qPCR?/U6	NS	38+/−4	45/40	Placenta	miR-95 and miR-548 upregulatedmiR-1246 downregulated	Correlated with serum lipids and adipokines, also with placental GLUTs
Zhao 2014 [82]	miR-518d	qPCR/snRNA U6	2 h, 75 g OGTT: fasting glucose > 5.6 mmol/L or 2 h > 8.6 mmol/L	37–40	40/40	Placenta	Upregulated in GD. PPAR-α is a predicted and validated target, with inverse placental protein expression	miRNA selected as placental marker
Yan 2018 [80]	Circular RNA profiling	NGS (Illumina HiSeq)+qPCR/GAPDH	NS	38–41	30/30	Placenta	From a total of 48,270 circRNAs, 227 were upregulated and 255 down-regulated	Enrichment of pathways involved in glucose and lipid metabolism
**Other tissues**								
Floris 2015 [64]	miR-101	qPCR/SnU6B	At 24–28 weeks’ gestation with fasting glycemia >95 mg/dL and >155 mg/dL two hours after a 75 g OGTT	Term (NS)	18/18	HUVEC	Increased expression in HUVECs of GD, which affects their survival and functional capabilities	Mir101 selected due to relationship with endothelial function and angiogenesis
Tryggestad 2016 [77]	miR-30c-5p miR-452-5p miR-126-3p miR-130b-3p miR-148a-3p miR-let-7a-5p miR-let-7g-5p	Array+qPCR on 32 miRNA/RNU 48	American Diabetes Association criteria [9] (A1 and A2)	NS	7/12	HUVEC	Among 19 detectable by qPCR, 7 upregulated in offspring of GD	Functional studies show decreased expression of AMPKa by transfection of miR-130 and miR-148a. AMPKa is known to stimulate glucose uptake and fatty acid oxidation
Shi 2014 [73]	miRNA expression profiling	AFFX miRNA expression chips +qPCR/miR-16	American Diabetes Association criteria (A1) [9]	38–39 (Caesarean section)	13/13	Omental adipose tissue	miR-222 upregulated in GD	One of 17 differentially expressed miRNAs.
Houshmand-Oeregaard 2018 [66]	mirR-15a miR-15b	qPCR/RNU48	NS. Screening performed in high-risk women	NA	76/42	Muscle of adult offspring of women with GD	Both miRNAs upregulated. Expression was correlated with personal and maternal glucose	miRNAs selected based on previous results. Involved in insulin secretion and resistance.

NS: Not specified. GA: Gestational age at sampling. Wk: weeks. NA: Not applicable. OGTT: Oral Glucose Tolerance Test. * Five per group for discovery array and 10 per group for qPCR. $ four per group in the discovery array and rest in the validation qPCR. ? not clear if the method is used. IADPG: International Association of Diabetes in Pregnancy Group. HUVEC: Human umbilical vein endothelial cells. NGS: Next generation sequencing. qPCR: quantitative polymerase chain reaction.

**Table 3:** Studies assessing the effect of pre-gestational diabetes on placental and offspring miRNAs.

Author Year [Reference]	Species	miRNA	Target Gene	Methods	Tissue	Results	Comments
Dong 2016 [87]	Mice	149 miRNAs	2111 potential target genes	RNA microarrays/RT-qPCR	Heart	Cardiac development-related pathways (STAT3 and IGF-1) and transcription factors associated to altered miRNAs, leading to CHDs	Oxidative stress as responsible for dysregulation of miRNAs
Dong D 2016 [88]	Mice	miR-17 downregulated	Thioredoxin interactive protein upregulated	RT-qPCR	Neural stem cells	Proapoptotic hyperglycaemia (via ASK1 pathway)	ASK1 leads to NTDs
Ibarra 2018 [85]	Human	miR-125-5p miR-20a-5p		RNA-Seq/qPCR	Placenta	Classifiers composed by 2–3 miRNAs were identified	miR-125-5p and miR-20a-5p were present in classifiers for type 1 and type 2 diabetes
Jiangs 2017 [89]	Human	miR130b-3p upregulated	*PGC-1* downregulation	RT-qPCR	Placental trophoblastic cell line (Be Wo cells)	Impaired mitochondrial function and oxidative stress which affects foetal development	Inhibition of miR-130b-3p reverted effects found.
Ramya, 2017 [90]	Mice	miRNA-30 family miR-30b upregulated	Sirtuin gene downregulated	RNA microarrays/RT-qPCR	Neural stem cells	Decreased Sirt 1 protein: altered neuron/glia ratio	Diabetic induced NTDs via miRNAs
Shi 2017 [91]	Mice	Exosomal miRNA		RNA-Seq analysis	Blood	Maternal exosomal miRNAs in diabetes contribute to cardiac development deficiency leading to CHDs	Maternal exosomal miRNAs in diabetes could cross the maternal-foetal barrier
Shyama sundar 2013 [92]	Mice	miR-200a, miR-200b, miR-466a-3p, miR-466d-3p Downregulated	*Dcx* and *Pafah1b1* upregulated	RT-qPCR	Neural stem cells	Knock down of miRs increases gliogenesis and neurogenesis which if impaired may form the basis of NTDs	Hyperglycaemia alters epigenetic-reversible mechanisms in NSCs.
Wang 2017 [93]	Mice	miR192-2 upregulated	*PGC-1* gene upregulated	RT-qPCR	Neuroepithelial cells	Less NTDs by diminishing autophagy	These regulate the teratogenicity of hyperglycaemia
Zhao 2017 [94]	Mice	miR-505-5p, miR-770-5p and miR-1a-1-5p differentially expressed	Association with diabetic embryopathy was sought	NGS	Embryos (9.5 days)	Putative target genes under-represented in a database of genes associated with cardiovascular and neural malformations	No differences in miRNA expression at 8.5 days
Zhao 2018 [95]	Mice	miR-27a upregulated	Nuclear factor erythroid 2-related factor 2 downregulated	RT-qPCR	Neural stem cells	Increased oxidative stress that suppresses Nuclear factor erythroid 2-related factor 2 and its responsive antioxidant enzymes resulting in diabetic embryopathy	Protein reduction also followed a (glucose) dose and time dependent-manner

C: control. CHDs: Congenital heart defects. D: diabetes. N: number. NS: Not specified. NSC: neural stem cells. NTDs: Neural tube defects. CHD: Congenital heart defects. NGS: Next generation sequencing. RT-qPCR: Real-time, quantitative PCR.
